# RailTrack-DaViT: A Vision Transformer-Based Approach for Automated Railway Track Defect Detection

**DOI:** 10.3390/jimaging10080192

**Published:** 2024-08-07

**Authors:** Aniwat Phaphuangwittayakul, Napat Harnpornchai, Fangli Ying, Jinming Zhang

**Affiliations:** 1 International College of Digital Innovation, Chiang Mai University, Chiang Mai 50200, Thailand; aniwat.ph@cmu.ac.th (A.P.); jinming.z@alumni.cmu.ac.th (J.Z.); 2 Lancang-Mekong Digital Intelligence (Shijiazhuang) Technology Research Center, Shijiazhuang 051230, China; 3 Faculty of Economics, Chiang Mai University, Chiang Mai 50200, Thailand; 4 State Key Laboratory of Bioreactor Engineering, Department of Computer Science and Engineering, East China University of Science and Technology, Shanghai 200237, China; yfangli@ecust.edu.cn

**Keywords:** railway track inspection, vision transformer, computer vision, transportation safety, public transportation monitoring

## Abstract

Railway track defects pose significant safety risks and can lead to accidents, economic losses, and loss of life. Traditional manual inspection methods are either time-consuming, costly, or prone to human error. This paper proposes RailTrack-DaViT, a novel vision transformer-based approach for railway track defect classification. By leveraging the Dual Attention Vision Transformer (DaViT) architecture, RailTrack-DaViT effectively captures both global and local information, enabling accurate defect detection. The model is trained and evaluated on multiple datasets including rail, fastener and fishplate, multi-faults, and ThaiRailTrack. A comprehensive analysis of the model’s performance is provided including confusion matrices, training visualizations, and classification metrics. RailTrack-DaViT demonstrates superior performance compared to state-of-the-art CNN-based methods, achieving the highest accuracies: 96.9% on the rail dataset, 98.9% on the fastener and fishplate dataset, and 98.8% on the multi-faults dataset. Moreover, RailTrack-DaViT outperforms baselines on the ThaiRailTrack dataset with 99.2% accuracy, quickly adapts to unseen images, and shows better model stability during fine-tuning. This capability can significantly reduce time consumption when applying the model to novel datasets in practical applications.

## 1. Introduction

Railways contribute significantly to economic growth, especially in developing countries [1]. Moreover, railway transportation systems are a more environmentally friendly mode, with less noise pollution and energy consumption compared to airways, roads, and waterways [2,3]. Railway tracks play a crucial role in ensuring the safe and efficient operation of railway transportation [4]. However, railway track defects are a common cause of train accidents, which can lead to injuries, fatalities, and significant economic losses. Therefore, the regular inspection of railway tracks is essential to identify and address potential defects [5]. Traditional railway track inspection methods involve visual and manual inspection by human experts or using specialized vehicles equipped with sensors. However, these methods are time-consuming, costly, and may not always be effective at detecting defects [6,7].

There are alternative methods to detect damage and monitor the railway tracks. Loveday [8] explores a method to detect axial stress in railway tracks by using guided waves. Hashmi et al. [9] replaced the manual fault identification on the railway, which is performed by a railway engineer with on-the-fly feature extraction. This feature extraction is based on a deep learning model to generate spectrograms that can ensure they are less time-consuming and prone to error than traditional systems. Three deep learning models, including Convolutional 1D, Convolutional 2D, and Long-Short Term Memory (LSTM), are used to analyze different lengths of audio samples.

Computer vision is another technique that can be applied to railway track inspection and monitoring. There are a number of approaches proposed for inspecting and monitoring railway tracks using computer vision techniques. Ruvo et al. [10] present a Visual Inspection System for railway maintenance that can detect and track the rail head in a video sequence, reducing the area to be analyzed by using FPGA technology, which is highly flexible and configurable as it is based on classifiers that can be easily reconfigured for different types of rails. Ritika and Rao [11] propose a method to detect track anomalies, such as vegetation overgrowth and sun kinks, using a camera mounted on a moving locomotive and a simulated image pipeline. The Inception V3 model is used for the binary classification of vegetation overgrowth and sun kinks, and the trained model is tested on professionally simulated track videos. Although the model shows that the proposed method can classify track anomalies with high precision, the method relies on a camera mounted on a moving locomotive, which may not be feasible in all situations or for all types of tracks. Moreover, the simulated images used for training the model may not perfectly represent real-world track anomalies, which could affect the accuracy of the model. Gasparini et al. [12] proposed a vision-based framework for detecting obstacles on railways during the night using RGB or thermal images. The framework uses a rail drone equipped with cameras and external light sources to collect data. The collected data are used to train a deep learning model that can accurately detect obstacles. The experiments show that the proposed approach is suitable for implementation on a self-powered drone. Nonetheless, the use of drones to collect data is limited by their unpredictable position and short battery life. Gasparini et al. [13] proposed another framework for the automatic inspection of railways during the night using thermal images. The framework consists of three modules for detecting, localizing, and classifying anomalies. The authors also introduce a new dataset called Vesuvio, which was acquired from a rail drone specifically created for anomaly detection tasks in a railway scenario during the night. However, the power consumption of light sources used for night vision is limited to rail drones that are self-powered. Acquisition cameras with a high spatial resolution are needed to detect even small-sized objects. Gibert et al. [14] proposed a new method for fastener detection in railway tracks using a combination of linear Support Vector Machine (SVM) classifiers and a histogram of oriented gradients features to improve the classification margin. The system can inspect ties for missing or defective rail fastener problems for missing and broken components with grayscale images. Even though these two methods show high accuracy for classification, they are only applicable for grayscale image datasets. In 2016, Gibert et al. [15] proposed an algorithm for the automated inspection of railway tracks using deep convolutional neural networks and a multi-task learning framework to classify different natural color images of materials and fasteners. The proposed algorithm achieved high accuracy in detecting fasteners and ties, demonstrating its effectiveness in detecting different types of fasteners and materials. However, the proposal for the method does not discuss the computational requirements, which could be a limitation in practical applications. Liu et al. [16] present a method to improve the performance of rail fastener defect inspection for multiple railways to ensure the safety of railway operation, including a fastener region location method based on an online learning strategy and a fastener defect recognition method based on a deep convolutional neural network. One limitation of the proposed method is that the increase in the maximum queue length of the online template library improves the detection rate but reduces the detection speed, thus affecting the system’s efficiency. Eunus et al. [4] introduced a novel Deep Learning (DL) algorithm named ECARRNet for automatic fault detection in railway tracks. This method aims to reduce accidents on railway tracks, save lives, and prevent disasters.

All the above works rely on Convolutional Neural Networks (CNNs). However, CNNs struggle with capturing long-range dependencies due to their local receptive field limitations [17]. Images with thin structures, complex spatial layouts, or multiple views tend to exhibit long-range dependencies [18,19]. Capturing long-range dependencies is crucial for the tasks that handle the complex and interconnected-structure images [20]. Vision Transformer (ViT) [21] is one method that can effectively handle this type of image. Vision Transformers are currently applied to various visual inspection tasks such as road tunnel defect classification [22] and structural condition assessment [23]. In this study, we employ Vision Transformer instead of CNNs for the railway track inspection task. ViT is a deep learning model architecture that is designed for image recognition tasks. Traditional CNN uses a set of convolutional filters to extract image features, which are then processed by fully connected layers to produce a final prediction. ViT replaces the convolutional layers of CNN with a self-attention [24] mechanism. It processes the input image directly by dividing an image into a sequence of non-overlapping patches of equal size. Then, each patch is embedded linearly in a low-dimensional feature space. These embedded patches form the input sequence for Transformer blocks. In the Transformer blocks, the self-attention mechanism is used to monitor all input patches and capture their dependencies. The output of the self-attention mechanism is then passed through a feedforward neural network to produce the block’s final output. The output of the last block is then fed to a classification head, which produces the final prediction. In addition to patch embeddings, ViT includes positional embeddings that provide information about the spatial location of each patch. This enables the model to encode the spatial relationships between patches in an image and capture the long-range dependencies, enabling stronger global context modeling [25,26,27]. Additionally, ViT can handle images of any size because it processes image patches independently. This makes ViT more adaptable than CNNs, which require the image to be resized or cropped to a fixed size. Furthermore, ViT has shown promising results not only on large datasets but also on small datasets [28]. Moreover, ViT has demonstrated state-of-the-art (SOTA) performance on a variety of image classification tasks, outperforming CNNs on certain benchmark datasets [21].

This paper proposes a novel transformer-based deep learning approach, called RailTrack-DaViT, for detecting defects in railway track images. Railway track images inherently contain long-range dependencies due to the extended, curved geometry of the tracks themselves. Capturing global context and relationships between distant regions is crucial for the classification task. The key contributions are listed as follows:

- RailTrack-DaViT is a novel approach that applies a Vision Transformer (ViT) to railway track defect binary classification. RailTrack-DaViT is not only able to capture long-range dependencies but also effectively models both global and local information in railway track images.
- RailTrack-DaViT incorporates a customized classification head and training pipeline that can be effectively trained and tested on datasets containing limited data. These modifications enable RailTrack-DaViT to achieve greater stability and quicker adaptation to unseen images during the fine-tuning process compared to baseline methods.
- The ThaiRailTrack dataset is constructed by collecting Thai railway track images from two sources: the National Science and Technology Development Agency (NSTDA) and the Passenger Service Department (Operation) of the State Railway of Thailand.
- A comprehensive analysis of model performance is presented, including metrics such as a confusion matrix, training history visualization, and classification metrics. For extensive evaluation, the proposed model is evaluated on various datasets, including Rail, Fastener and fishplate, Multi-faults, and ThaiRailTrack datasets.

## 2. Method

### 2.1. Overview of RailTrack-DaViT Architecture

**Figure 1:**
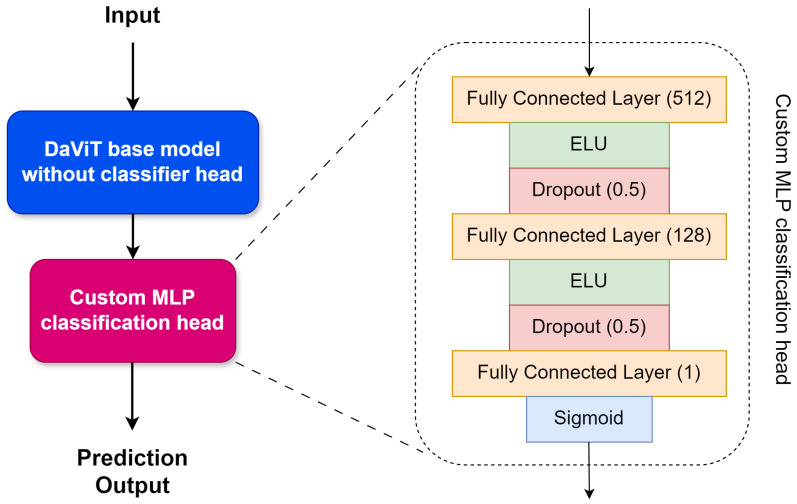
RailTrack-DaViT network architecture.

In this study, we propose a deep learning approach for defecting defects in railway tracks, called RailTrack-DaViT. The RailTrack-DaViT utilizes a pre-trained Dual Attention Vision Transformers (DaViT) [29] base model as the feature extractor backbone. DaViT is a recently proposed vision transformer variant that achieves a strong performance on ImageNet [30] classification while using fewer parameters and demonstrating improved data efficiency compared to other Vision Transformer (ViT) models [29] architectures. With spatial window and channel group attentions, DaViT efficiently captures not only local representation but also global context. To leverage the powerful representations learned by DaViT on ImageNet while reducing the risk of overfitting to our smaller railway dataset, we froze all layers of the base model initially and used it as a fixed feature extractor. The original 1000-class classification head was replaced with a custom multilayer perceptron (MLP) classification head for a binary classification task. The completed network architecture of RailTrack-DaViT is shown in Figure 1.

The MLP classification head consists of two fully connected layers with 512 and 128 units, respectively. The ELU [31] activation functions were utilized for non-linearity as shown in Equation (1).

(1) $$f\left(z\right)=\left\{\begin{array}{cc} z & \mathrm{for}\;z\geq 0\\ \beta \cdot (e^{z}-1) & \mathrm{for}\;z\leq 0 \end{array}\right.$$

where:

- *z* is the feature between layers used as input for the ELU activation function;
- β is a hyperparameter that controls the value to which an ELU saturates for negative inputs.

ELU has been shown to speed up learning in deep neural networks and tends to have a significantly better generalization performance compared to other activation functions. Moreover, dropout regularization (p=0.5) between each layer was applied. The final output is a single sigmoid unit representing the probability of a defect being present in the input image.

### 2.2. RailTrack-DaViT Model Training Process

**Figure 2:**
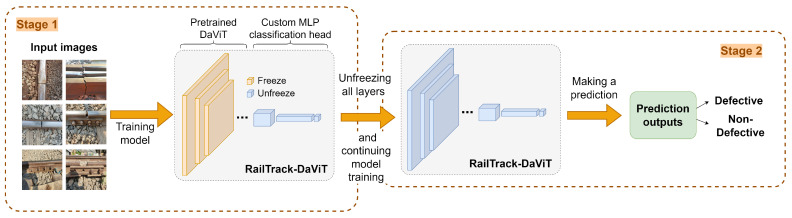
Overall process for training RailTrack-DaViT.

Figure 2 illustrates the overall process in training RailTrack-DaViT. The RailTrack-DaViT was trained in two stages. In the first stage, only the classification head was trained for 90 epochs while the RailTrack-DaViT backbone remained frozen. Binary cross-entropy loss was used as the loss function. The equation of binary cross-entropy loss can be derived as:

(2) $$L\left(y,\hat{y}\right)=-\frac{1}{N}\sum_{i=1}^{N}\left[y_{i}\log\left({\hat{y}}_{i}\right)+\left(1-y_{i}\right)\log\left(1-{\hat{y}}_{i}\right)\right]$$

where:

- $L(y,\hat{y})$ is the binary cross-entropy loss;
- *N* is the number of samples;
- $y_{i}$ is the true label of the *i*-th sample (0 or 1);
- ${\hat{y}}_{i}$ is the predicted probability of the *i*-th sample belonging to class 1.

The loss function penalizes the model for making incorrect predictions. It encourages the predicted probabilities to be close to the true labels, minimizing the difference between them. The logarithmic terms ensure that the loss is high when the predicted probability is far from the true label and low when it is close.

The optimization was performed using the Adam optimizer [32] with a learning rate of 0.001. The one-cycle learning rate policy [33] was employed and linearly increased the learning rate from a low value to a maximum of 0.003 in the first 30% of iterations, then linearly decreased it back to the minimum in the remaining iterations. The one-cycle learning rate policy can be expressed as Equation (3). This learning rate schedule has been shown to speed up convergence and achieve better final accuracy compared to a fixed learning rate.

(3) $$\alpha \left(t\right)=\left\{\begin{array}{cc} \alpha_{\min}+\frac{t}{0.3T}\left(\alpha_{\max}-\alpha_{\min}\right), & 0\leq t<0.3T\\ \alpha_{\max}-\frac{t-0.3T}{0.7T}\left(\alpha_{\max}-\alpha_{\min}\right), & 0.3T\leq t\leq T \end{array}\right.$$

where:

- α(t) is the learning rate at iteration *t*;
- $\alpha_{\min}$ is the minimum learning rate (initial learning rate, $1\times 10^{-3}$);
- $\alpha_{\max}$ is the maximum learning rate ($3\times 10^{-3}$);
- *T* is the total number of iterations (steps per epoch × number of epochs).

In the second stage, the entire RailTrack-DaViT model was unfrozen and fine-tuned end-to-end for an additional 10 epochs. The model was trained during these 10 epochs by utilizing the Adam optimizer with an initial learning rate of 0.0001 and a one-cycle learning rate policy with a maximum learning rate of 0.001. This fine-tuning allows the visual features extracted by RailTrack-DaViT to adapt to specific defect patterns present in the dataset, while the lower learning rate helps limit overfitting.

To further improve training stability, gradient clipping [34] with a maximum L2 norm regularization of 1.0 was applied to the gradients at each optimization step. Gradient clipping is a technique that limits the magnitude of gradients to prevent unstable training. It prevents the gradients from growing too large and causing training to diverge. It is typically formulated as

(4) $$g_{\mathrm{clipped}}=\frac{g}{\max\left(1,\frac{{∥g∥}_{2}}{c}\right)}$$

where:

- g is the gradient vector;
- ${∥g∥}_{2}$ is its L2 norm;
- *c* is the clipping threshold.

The clipping threshold value *c* determines the maximum allowed L2 norm of the gradient. In this study, we set the clipping threshold equal to 1.0. This means that if the gradients have an L2 norm larger than 1.0, they will be clipped to have an L2 norm of 1.0 while preserving direction. Gradients with an L2 norm less than or equal to 1.0 will remain unchanged.

## 3. Dataset

**Figure 3:**
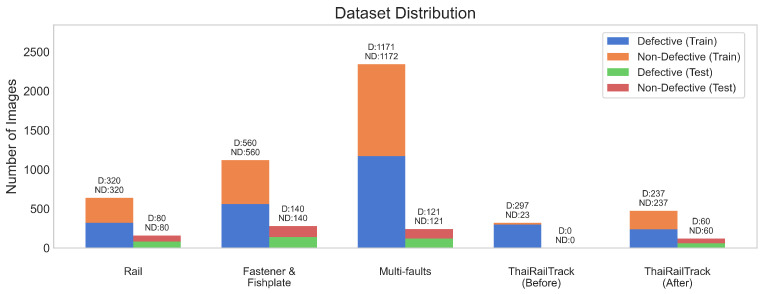
The number of defective and non-defective images in both the training and test sets of Rail, Fastener and fishplate, Multi-faults, and ThaiRailTrack datasets. “D” refers to the defective images category and “ND” refers to the non-defective images category.

There are four datasets that are utilized to evaluate the performance of our approach in this work. The datasets consist of Rail, Fastener and fishplate, Multi-faults, and ThaiRailTrack datasets. The first three datasets (Rail, Fastener and fishplate, and Multi-faults datasets) are public datasets that can be accessed through Kaggle (https://www.kaggle.com/datasets (accessed on 19 April 2024)), which is an online community platform for data scientists and machine learning practitioners. These datasets were constructed by Eunus et al. [4]. ThaiRailTrack dataset is a private dataset that is collected from Thailand’s railway organizations. Figure 3 presents the distribution of images of railway track defect detection across the four datasets used in our study. ThaiRailTrack (Before) represents the number of images before oversampling. ThaiRailTrack (After) presents the number of images after performing the oversampling technique.

The details of each dataset are described as follows:

### 3.1. Rail Dataset

**Figure 4:**
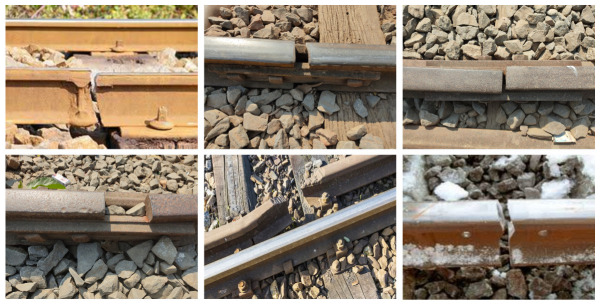
The sample images of railway tracks with faults (defect). The dataset consists of images of different views, including top and side views.

The rail dataset [35] contains images of regular railway tracks and faults on railway tracks. There are 800 defective and non-defective images. The dataset was divided into 640 images for the training set and 160 images for the test set. Examples of defective images on rails are illustrated in Figure 4.

### 3.2. Fastener and Fishplate Dataset

**Figure 5:**
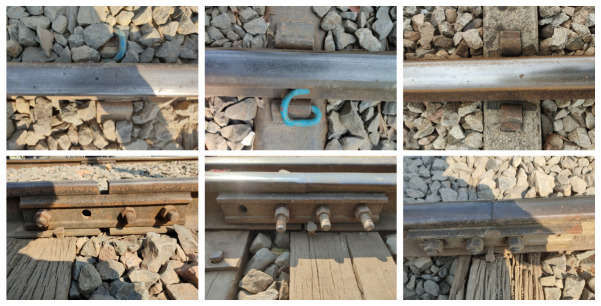
The sample images of broken fasteners and fishplates. The dataset consists of images from different views including top and side views. The first row presents the images of broken fasteners on railway tracks. The second row represents the images of broken fishplates on railway tracks.

The Fastener and fishplate dataset [36] contains images of regular railway tracks, broken clips (fasteners), and broken fishplates. Clips are a type of fastener responsible for securing the steel rail to the sleeper. Occasionally, the clip may fracture or become detached, making it incapable of serving its intended function [37]. Rail fishplates, one of the most common types of rail fastener [38], are often small copper or nickel silver plates employed to connect two rails together in a railway track. They ensure the proper alignment and continuity of the rails. In total, there were 1300 images of defective and non-defective fasteners and fishplates. The images were divided into 1120 for the training set and 280 for the test set. The samples of broken fasteners and fishplates on railway tracks are illustrated in Figure 5.

### 3.3. Multi-Faults Dataset

The Multi-faults dataset is a combination of three datasets—Rail [35], Fastener and fishplate [36], and Railway Track fault Detection [39] datasets. There are 2584 images of both defective and non-defective railway tracks. The images were split into 2343 for the training set and 242 for the test set.

### 3.4. ThaiRailTrack Dataset

**Figure 6:**
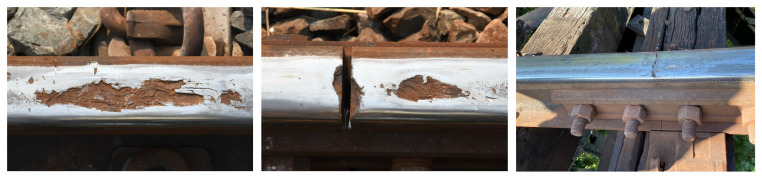
The top and side image views of Thai railway tracks collected from two Thailand organizations, NSTDA and the Passenger Service Department (Operation) of the State Railway of Thailand.

The ThaiRailTrack dataset consists of railway track images in Thailand, including both defective and non-defective tracks collected from two sources—the National Science and Technology Development Agency (NSTDA) and the Passenger Service Department (Operation) of the State Railway of Thailand. The original dataset had 297 images of defective railway tracks and 23 of non-defective railway tracks. To prevent a class imbalance problem when evaluating the model, we applied an oversampling technique by using random data augmentation for images of non-defective tracks. Finally, we obtained 594 images. The images were divided into 474 images for the training set and 120 images for the test set. The example images from the ThaiRailTrack dataset are illustrated in Figure 6.

## 4. Experiment and Result Analysis

### 4.1. Performance Confusion Matrix

**Figure 7:**
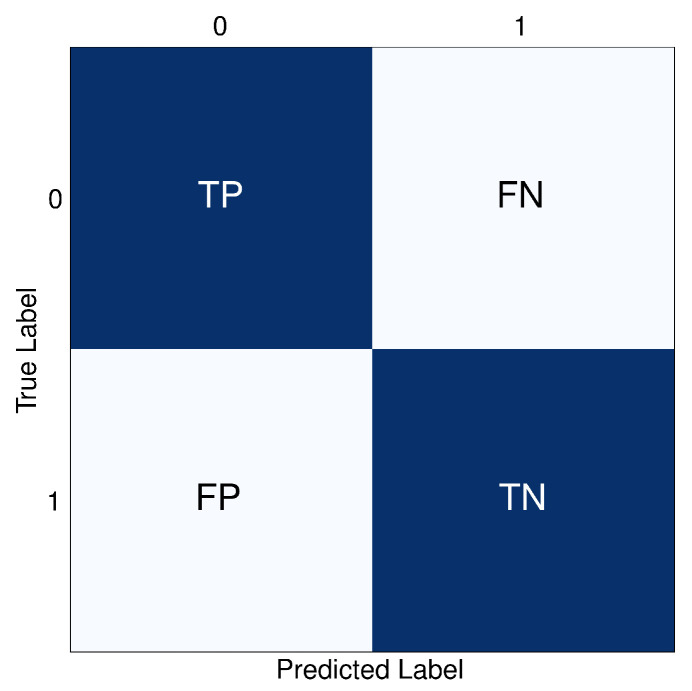
The confusion matrix of binary classification consisting of x-axis, y-axis, and the values.

A confusion matrix is a table used to describe the performance of a classification model on a set of test data where the true values are known [40]. Figure 7 illustrates the confusion matrix for binary classification utilized in this study. The matrix consists of two axes—x-axis and y-axis—with corresponding labels. The 0 label represents positive or defective samples, which are images of broken rail tracks. The 1 label denotes negative or non-defective samples, which are images of regular rail tracks. TP, TN, FP, and FN refer to True Positive, True Negative, False Positive, and False Negative, respectively.

### 4.2. Performance Evaluation Metrics

In this study, we utilized key performance evaluation metrics for classification models. The metrics include precision, recall, specificity, F1 score, and accuracy. These key performance evaluation metrics can be calculated with the following equations:

Precision measures the proportion of true positive predictions among all positive predictions:

(5) $$\mathrm{Precision}=\frac{TP}{TP+FP}\times 100\%.$$

Recall or sensitivity measures the proportion of actual positives that are correctly identified by the model:

(6) $$\mathrm{Recall}=\frac{TP}{TP+FN}\times 100\%.$$

Specificity measures the proportion of actual negatives that are correctly identified by the model:

(7) $$\mathrm{Specificity}=\frac{TN}{TN+FP}\times 100\%.$$

The F1 score provides a balanced measure of a model’s performance, especially when the classes are imbalanced:

(8) $$F1\;\mathrm{score}=\frac{2\times Precision\times Recall}{Precision+Recall}\times 100\%=\frac{2\times TP}{2\times TP+FP+FN}\times 100\%$$

Accuracy measures the overall correctness of model’s predictions:

(9) $$\mathrm{Accuracy}=\frac{TP+TN}{TP+TN+FP+FN}\times 100\%.$$

### 4.3. Data Pre-Processing

In the beginning, we prepared four different datasets including Rail, Fastener and fishplate, Multi-faults, and ThaiRailTrack. The Rail, Fastener and fishplate, and ThaiRailTrack datasets were divided into 80 percent for the training set and 20 percent for the test set. For the Multi-faults dataset, we used 10 percent as the test set. Four types of data augmentation were randomly applied, including horizontal rotation, vertical rotation, 90 or 270-degree rotations, and brightness adjustment during model training. The images were performed randomly center-cropped to 256×256, resized to 224×224, and then randomly shuffled with a seed value equal to 42 before model training. In this study, we set the batch size to 16.

### 4.4. Comparison Models Overview

In this study, there are different CNN-based models as the baseline, compared with our RailTrack-DaViT for image classification. The models include Xception [41], ResNet-18 [42], ResNet-50 [42], EfficientNet-B0 [43], and EfficientNet-B7 [43]. The models were implemented using the PyTorch library in Python and executed on an NVIDIA GeForce RTX 3060 GPU. To ensure a fair comparison, we established consistent parameters for training both RailTrack-DaViT and the baseline models. The training parameters were set as follows: learning rate of 0.001, Adam optimizer, and batch size of 16.

Xception [41]: A deep convolutional neural network architecture inspired by Inception [44]. It replaces the standard Inception modules with depthwise separable convolutions, which results in a more efficient use of model parameters.

ResNet or Residual Networks [42]: The key innovation of ResNet is the introduction of “identity shortcut connections” that skip one or more layers, allowing the network to learn residual functions with reference to the layer inputs. ResNet-18 and ResNet-50 are two specific architectures with 18 and 50 layers, respectively.

EfficientNet [43]: A family of models that are designed to achieve better accuracy and efficiency. The key idea is to uniformly scale the network width, depth, and resolution with a set of fixed scaling coefficients. EfficientNet-B0 is the baseline model and EfficientNet-B7 is a larger model obtained by scaling up the baseline.

### 4.5. The Performance Evaluation of RailTrack-DaViT and Conventional CNN-Based Models on Rail Dataset

**Figure 8:**
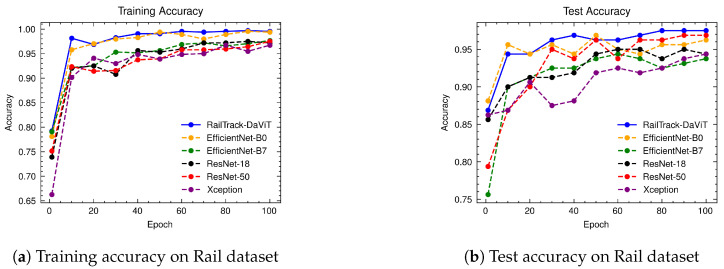
Comparative analysis of training and test accuracies for different classification models on the Rail dataset over 100 training epochs.

Figure 8 illustrates the training and test accuracy of baseline and RailTrack-DaViT on the Rail dataset. The models were trained on the Rail dataset with 100 epochs. In terms of training accuracy, RailTrack-DaViT achieved the highest accuracy from the early epoch. It achieved peak accuracy compared to other models in five epochs and constantly increased the accuracy up to 100 epochs. Additionally, RailTrack-DaViT performed the highest test accuracy, which was evaluated on the test set of the rail dataset after training the model for 30 epochs. The performance curves for precision, recall, specificity, and F1 score of the baseline models and RailTrack-DaViT on the Rail dataset are presented in Appendix A.

Table 1 demonstrates that RailTrack-DaViT and ResNet-50 achieve an impressive performance across all evaluation metrics tested on the Rail dataset. Specifically, for the “Defective” category, it attains a precision of 96.3%, a recall of 97.5%, a specificity of 96.3%, and an F1 score of 96.9%. The overall average accuracy of RailTrack-DaViT is 96.9%, which is among the highest in the table, indicating its exceptional performance in defect classification.

**Figure 9:**
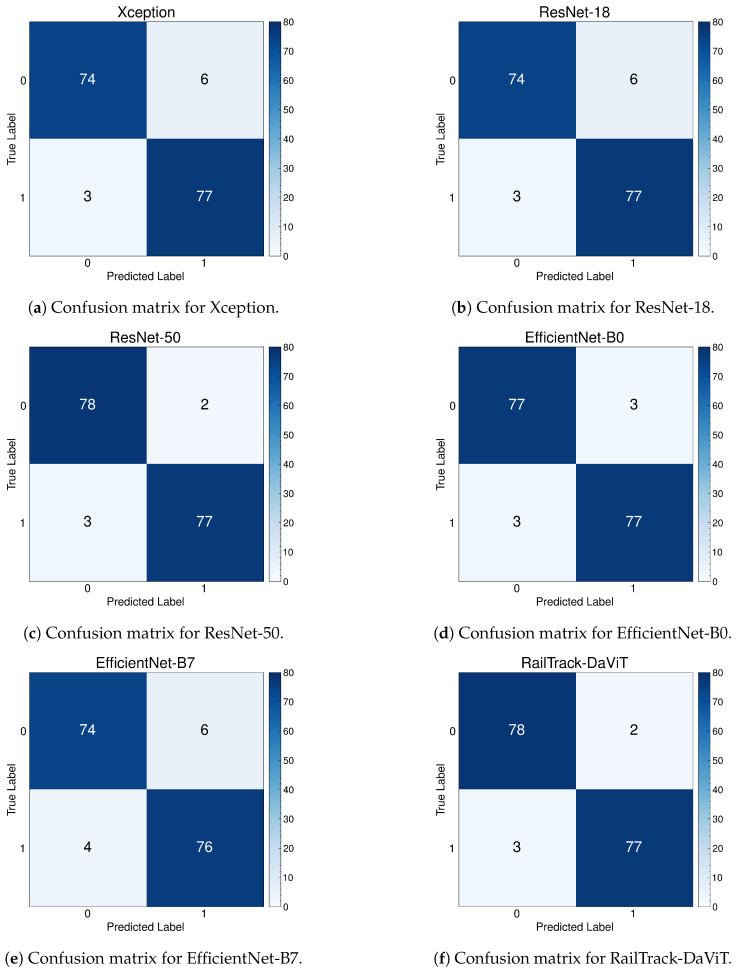
Confusion matrices for baseline and our RailTrack-DaViT on the Rail dataset.

Figure 9 represents the confusion matrices for the baseline and our proposed model on the test set of the Rail dataset. The results show that RailTrack-DaViT and ResNet-50 can produce the highest true positives and true negatives, which equal 78 and 77, respectively, while preserving the lowest false negatives and false positives, which equal 2 and 3, respectively. This demonstrates that our proposed model is comparative to the traditional CNN-based baseline model for this Rail dataset.

### 4.6. The Performance Evaluation of RailTrack-DaViT and Conventional CNN-Based Models on Fastener and Fishplate Dataset

**Figure 10:**
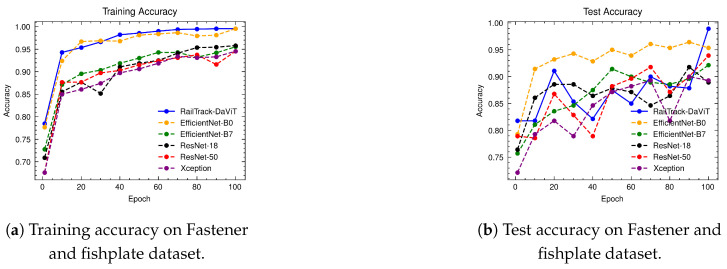
Comparative analysis of training and test accuracies for different classification models on Fastener and fishplate dataset over 100 training epochs.

Figure 10 presents a comprehensive analysis of the training and test accuracy of CNN-based deep learning models and our RailTrack-DaViT over 100 training epochs. The RailTrack-DaViT model consistently outperforms other models in training accuracy. Despite fluctuations in test data performance due to the optimization landscape of the Transformer-based model itself or dataset complexities arising from a combination of fastener and fishplate images, the accuracy significantly improves after unfreezing the model’s weights (the last 10 epochs). This demonstrates the impact of unfrozen layer operations on model efficiency. The additional performance curves are presented in Appendix B.

Table 2 represents the performance metrics for CNN-based classification models and our proposed model on the Fastener and fishplate dataset. According to the table, the RailTrack-DaViT model demonstrates exceptional performance across both the defective and non-defective categories, with consistently high values for precision, recall, specificity, F1 score, and accuracy. The overall average metrics further underscore the robust and balanced performance of the model.

**Figure 11:**
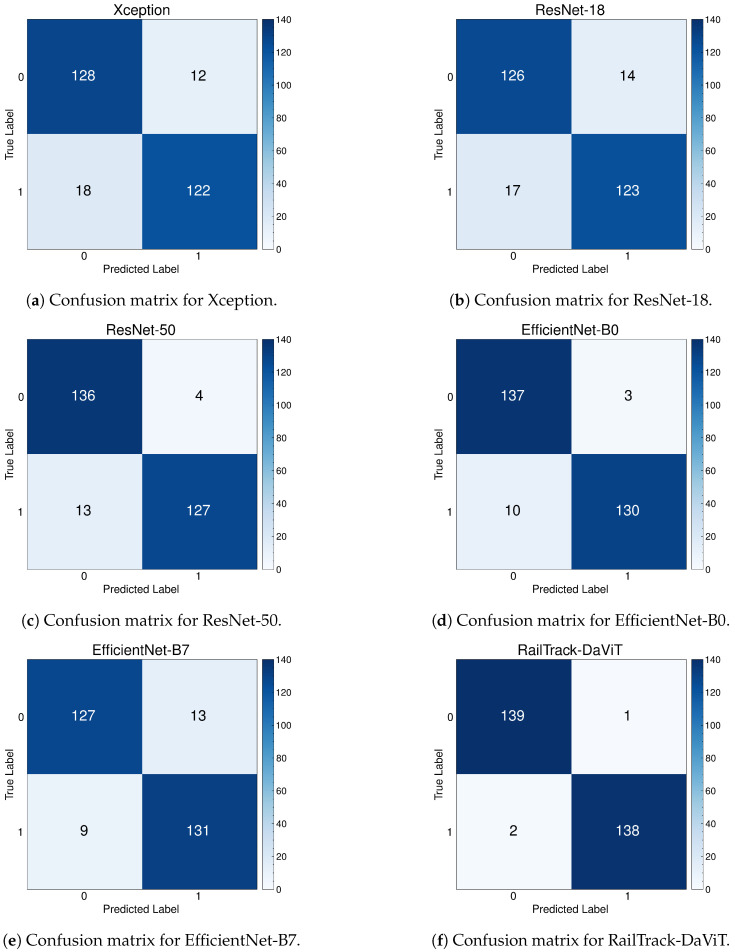
Confusion matrices for baseline and our RailTrack-DaViT on the Fastener and fishplate dataset.

Figure 11 represents the confusion matrices for baseline models and our proposed model on the test set of the Fastener and fishplate dataset. Based on 280 images of the test set, the confusion matrix reveals that the RailTrack-DaViT outperforms the other models by correctly classifying between defective and non-defective images with the highest true positives and true negatives.

### 4.7. The Performance Evaluation of RailTrack-DaViT and Conventional CNN-Based Models on Multi-Faults Dataset

**Figure 12:**
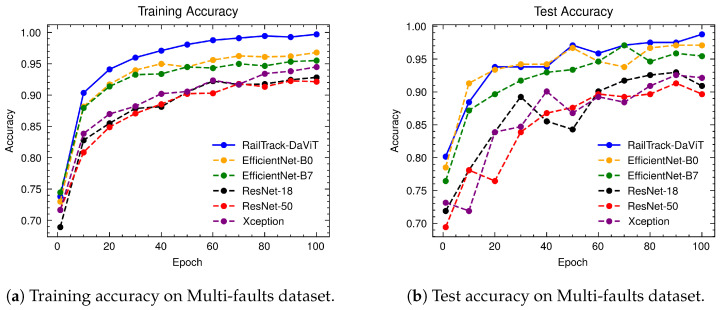
Comparative analysis of training and test accuracies for different classification models on the Multi-faults dataset over 100 training epochs.

Figure 12 presents a comprehensive evaluation of the training and test accuracy for various CNN-based models and our RailTrack-DaViT model on the Multi-faults dataset. Based on the training and test accuracy curve, the RailTrack-DaViT model (represented by the blue line) exhibits a consistent and steady increase in accuracy as the number of epochs progresses. It achieves the highest training and test accuracy at the beginning of model training. The RailTrack-DaViT can maintain a competitive performance and outperform the EfficientNet-B0 model in the later stages of training according to the test accuracy. The performance curves for precision, recall, specificity, and F1 score of the baseline models and RailTrack-DaViT on the Multi-faults dataset are presented in Appendix C.

Table 3 presents a comprehensive performance analysis of several models on various evaluation metrics on the Multi-faults dataset. The RailTrack-DaViT model demonstrates an excellent performance in distinguishing between defective and non-defective instances, with high scores across all evaluation metrics. This indicates its robustness and reliability in the defect defection task.

**Figure 13:**
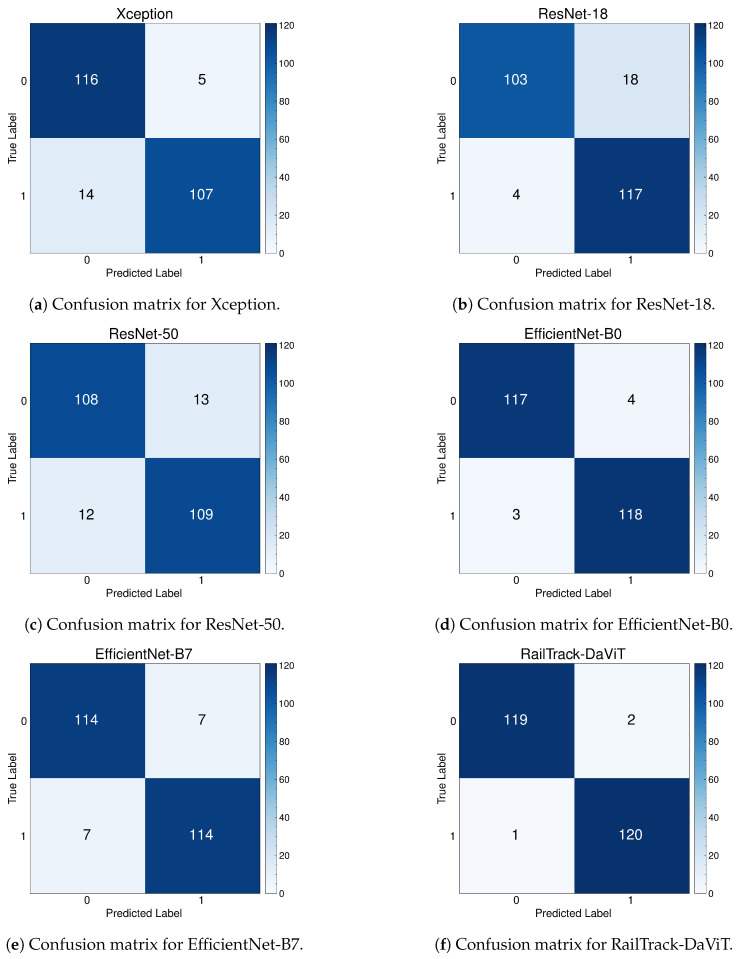
Confusion matrices for baseline and our RailTrack-DaViT on the Multi-faults dataset.

Figure 13 presents a comprehensive evaluation of the classification performance of various models, including our RailTrack-DaViT model, through a series of confusion matrices. For the 242 images in the test set in the Multi-faults dataset, the confusion matrix demonstrates that the RailTrack-DaViT model achieves the highest number of true positives and true negatives, equaling 119 and 120, respectively.

### 4.8. The Performance Evaluation of RailTrack-DaViT and Conventional CNN-Based Models on ThaiRailTrack Dataset

**Figure 14:**
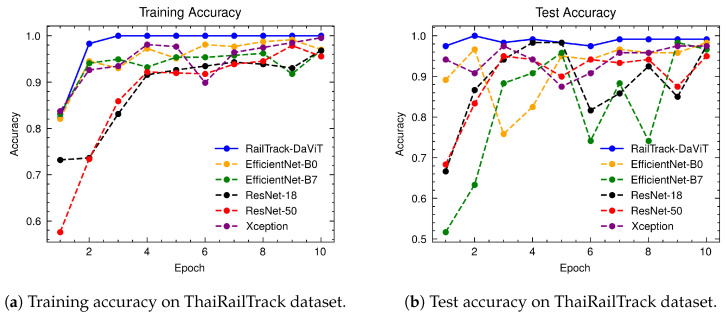
Comparative analysis of training and test accuracies for different classification models on ThaiRailTrack dataset over 10 training epochs.

Figure 14 presents a comparative analysis of the training and test accuracy of various models, including RailTrack-DaViT, over 10 epochs. It highlights the superior performance of RailTrack-DaViT in terms of both training and test accuracy, demonstrating its effectiveness in transfer learning and generalizing from the given data within only 10 epochs. Notably, the RailTrack-DaViT shows the highest test accuracy compared to baseline methods from the initial stages of fine-tuning. This suggests that the model adapts quickly to unseen images compared to other models. The performance curves for precision, recall, specificity, and F1 score of the baseline models and RailTrack-DaViT on the ThaiRailTrack dataset are presented in Appendix D.

Table 4 presents a comprehensive performance analysis of several models on the ThaiRailTrack dataset for various evaluation metrics. The RailTrack-DaViT achieves a remarkable performance across all metrics. Specifically, for the “Defective” category, it attains a perfect precision and specificity score of 100.0%, indicating no false positives and an excellent ability to identify true defective instances. Considering the average performance across categories, RailTrack-DaViT maintains its exceptional performance, achieving an average precision, recall, specificity, F1 score, and accuracy of 99.2%. This highlights the model’s robustness and ability to generalize effectively across diverse instances.

**Figure 15:**
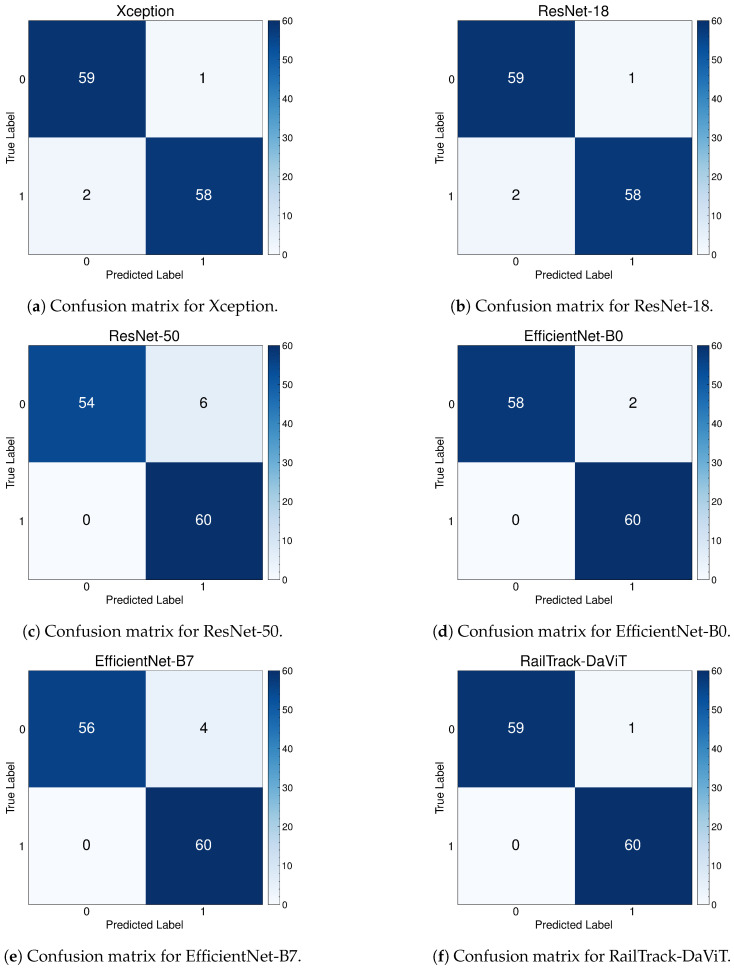
Confusion matrices for baseline and our RailTrack-DaViT on the ThaiRailTrack dataset.

Figure 15 presents a comprehensive evaluation of the classification performance of baseline and our proposed models through a series of confusion matrices. For the 120 images in the test set of the ThaiRailTrack dataset, the confusion matrix demonstrates that the RailTrack-DaViT model outperforms other models in terms of achieving the highest number of both true positives and true negatives. This indicates that our proposed model performs best on both defective and non-defective images.

## 5. Ablation Study

In this section, we analyze the impact of each optimizer, five-fold cross validation, and alternative network designs on the Multi-faults dataset. We focus on this dataset because it encompasses both the Rail dataset and the Fastener and fishplate dataset and contains the highest number of image samples.

### 5.1. Network Design

Table 5 presents the classification performance for each component and operation in the RailTrack-DaViT model, evaluated based on the training and test accuracies over 100 epochs. It demonstrates that RailTrack-DaViT without pre-trained weights achieves the lowest accuracies, indicating that the model fails to converge without pre-trained weights. Moreover, the results show that RailTrack-DaViT—with either an unfrozen layer operation or a custom MLP, or incorporating both—outperforms the traditional DaViT model.

### 5.2. Optimizer

**Figure 16:**
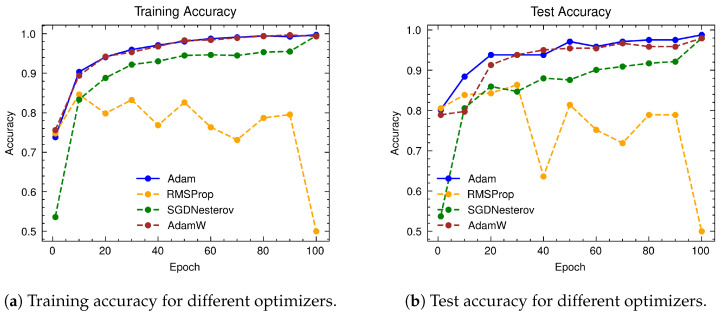
Comparative analysis of training and test accuracies for different optimizers on the Multi-faults dataset over 100 training epochs.

We selected the optimal optimizer based on its performance scores for both training and test sets. The RMSProp, SGD with Nesterov momentum, AdamW, and Adam optimizers were evaluated for model performance. Figure 16 illustrates the training and test accuracies of RailTrack-DaViT utilizing different optimizers. The Adam optimizer demonstrates the best performance and greater stability compared to the others on both training and test sets.

### 5.3. Five-Fold Cross Validation

In addition to standard model training, we conducted five-fold cross-validation to monitor the average performance accuracy metrics on both training and test sets. In this study, the training set was randomly split into *k* folds, where k=5. The model was then trained on k−1 folds, while one fold was left for model validation.

Table 6 summarizes the comparison of training and test accuracies between standard model training and five-fold cross-validation. Our RailTrack-DaViT model achieves the highest scores in both training and test accuracies while maintaining model training stability, as evidenced by the lowest standard deviation values (0.1 for training accuracy and 0.4 for test accuracy) among baseline models.

## 6. Discussion and Conclusions

In this paper, we proposed RailTrack-DaViT, a novel vision transformer-based deep learning approach for detecting defects from railway track images. By employing a Dual Attention Vision Transformer (DaViT) architecture, RailTrack-DaViT effectively captures both global and local information, addressing the limitations of traditional CNN-based models in capturing long-range dependencies on railway track datasets. The customized classification head and training pipeline enable the model to adapt pre-trained DaViT features for binary defect identification.

Extensive evaluations on various datasets, including Rail, Fastener and fishplate, Multi-faults, and ThaiRailTrack datasets, demonstrate the superior performance of RailTrack-DaViT compared to conventional CNN-based models used in this paper including Xception, ResNet-18, ResNet-50, EfficientNet-B0, and EfficientNet-B7. Overall, the proposed approach consistently achieves high precision, recall, specifically, F1 score, and accuracy across all datasets, highlighting its robustness and generalization capabilities. Moreover, when fine-tuning the model on the ThaiRailTrack dataset, RailTrack-DaViT demonstrates its capability for handing unseen data through its ability to quickly adapt to novel images. This rapid adaptation can significantly reduce time consumption in practical applications.

The ability of RailTrack-DaViT to capture long-range dependencies and effectively model both global and local information makes it a promising solution for automated railway track defect detection. By automating the inspection process, RailTrack-DaViT has the potential to significantly reduce the time and cost associated with manual inspections while improving the accuracy and reliability of defect detection. 

## Figures and Tables

**Table 1:** Classification report of various models for defective and non-defective images of the Rail dataset on different metrics.

Model	Category	Precision	Recall	Specificity	F1 Score	Accuracy
Xception	Defective	96.1	92.5	96.3	94.3	94.4
	Non-defective	92.8	96.3	92.5	94.5	94.4
	Average	94.4	94.4	94.4	94.4	94.4
ResNet-18	Defective	96.1	92.5	96.3	94.3	94.4
	Non-defective	92.8	96.3	92.5	94.5	94.4
	Average	94.4	94.4	94.4	94.4	94.4
ResNet-50	Defective	96.3	97.5	96.3	96.9	96.9
	Non-defective	97.5	96.3	97.5	96.9	96.9
	Average	96.9	96.9	96.6	96.6	96.9
EfficientNet-B0	Defective	96.3	96.3	96.3	96.3	96.3
	Non-defective	96.3	96.3	96.3	96.3	96.3
	Average	96.3	96.3	96.3	96.3	96.3
EfficientNet-B7	Defective	94.9	92.5	95.0	93.7	93.7
	Non-defective	92.7	95.0	92.5	93.8	93.7
	Average	93.8	93.7	93.7	93.7	93.7
RailTrack-DaViT	Defective	96.3	97.5	96.3	96.9	96.9
	Non-defective	97.5	96.3	97.5	96.9	96.9
	Average	96.9	96.9	96.9	96.9	96.9

**Table 2:** Classification report of various models for defective and non-defective images of the Fastener and fishplate dataset on different metrics.

Model	Category	Precision	Recall	Specificity	F1 Score	Accuracy
Xception	Defective	87.7	91.4	87.1	89.5	89.3
	Non-defective	91.0	87.1	91.4	89.1	89.3
	Average	89.4	89.3	89.3	89.3	89.3
ResNet-18	Defective	88.1	90.0	87.9	89.0	88.9
	Non-defective	89.8	87.9	90.0	88.8	88.9
	Average	88.9	88.9	88.9	88.9	88.9
ResNet-50	Defective	96.9	90.7	97.1	93.7	93.9
	Non-defective	91.3	97.1	90.7	94.1	93.9
	Average	94.1	93.9	93.9	93.9	93.9
EfficientNet-B0	Defective	93.2	97.9	92.9	95.5	95.4
	Non-defective	97.7	92.9	97.9	95.2	95.4
	Average	95.5	95.4	95.4	95.4	95.4
EfficientNet-B7	Defective	93.4	90.7	93.6	92.0	92.1
	Non-defective	91.0	93.6	90.7	92.3	92.1
	Average	92.2	92.1	92.1	92.1	92.1
RailTrack-DaViT	Defective	98.6	99.3	98.6	98.9	98.9
	Non-defective	99.3	98.6	99.3	98.9	98.9
	Average	98.9	98.9	98.9	98.9	98.9

**Table 3:** Classification report of various models for defective and non-defective images of the Multi-faults dataset on different metrics.

Model	Category	Precision	Recall	Specificity	F1 Score	Accuracy
Xception	Defective	89.2	95.9	88.4	92.4	92.1
	Non-defective	95.5	88.4	95.9	91.8	92.1
	Average	92.4	92.1	92.1	92.1	92.1
ResNet-18	Defective	96.3	85.1	96.7	90.4	90.9
	Non-defective	86.7	96.7	85.1	91.4	90.9
	Average	91.5	90.9	90.9	90.9	90.9
ResNet-50	Defective	90.0	89.3	90.1	89.6	89.7
	Non-defective	89.3	90.1	89.3	89.7	89.7
	Average	89.7	89.7	89.7	89.7	89.7
EfficientNet-B0	Defective	97.5	96.7	97.5	97.1	97.1
	Non-defective	96.7	97.5	96.7	97.1	97.1
	Average	97.1	97.1	97.1	97.1	97.1
EfficientNet-B7	Defective	94.2	94.2	94.2	94.2	94.2
	Non-defective	94.2	94.2	94.2	94.2	94.2
	Average	94.2	94.2	94.2	94.2	94.2
RailTrack-DaViT	Defective	99.2	98.3	99.2	98.8	98.8
	Non-defective	98.4	99.2	98.3	98.8	98.8
	Average	98.8	98.8	98.8	98.8	98.8

**Table 4:** Classification report of various models for defective and non-defective images of ThaiRailTrack dataset on different metrics.

Model	Category	Precision	Recall	Specificity	F1 Score	Accuracy
Xception	Defective	96.7	98.3	96.7	97.5	97.5
	Non-defective	98.3	96.7	98.3	97.5	97.5
	Average	97.5	97.5	97.5	97.5	97.5
ResNet-18	Defective	96.7	98.3	96.7	97.5	97.5
	Non-defective	98.3	96.7	98.3	97.5	97.5
	Average	97.5	97.5	97.5	97.5	97.5
ResNet-50	Defective	100.0	90.0	100.0	94.7	95.0
	Non-defective	90.9	100.0	90.0	95.2	95.0
	Average	95.5	95.0	95.0	95.0	95.0
EfficientNet-B0	Defective	100.0	96.7	100.0	98.3	98.3
	Non-defective	96.8	100.0	96.7	98.4	98.3
	Average	98.4	98.3	98.3	98.3	98.3
EfficientNet-B7	Defective	100.0	93.3	100.0	96.6	96.7
	Non-defective	93.8	100.0	93.3	96.8	96.7
	Average	96.9	96.7	96.7	96.7	96.7
RailTrack-DaViT	Defective	100.0	98.3	100.0	99.2	99.2
	Non-defective	98.4	100.0	98.3	99.2	99.2
	Average	99.2	99.2	99.2	99.2	99.2

**Table 5:** Accuracy of different network architectures and operations. The highest training and test accuracies are highlighted in bold.

Model	Pre-Training	Unfreeze	Custom Head	Training Accuracy	Test Accuracy
DaViT	✓	✗	✗	97.0	91.7
RailTrack-DaViT	✗	✗	✓	49.2	50.0
	✗	✓	✓	50.7	50.0
	✓	✗	✓	99.4	97.5
	✓	✓	✗	99.5	97.5
	✓	✓	✓	**99.7**	**98.8**

**Table 6:** Accuracy of various models using standard training and five-fold cross-validation on both training and test sets. “Standard” refers to standard model training, and “Five-fold” refers to five-fold cross-validation. The highest training and test accuracies are highlighted in bold.

Model	Technique	Training Accuracy	Test Accuracy
Xception	Standard	94.5	92.1
	Five-fold	93.5 ± 1.0	90.5 ± 1.3
ResNet-18	Standard	92.8	90.9
	Five-fold	93.3 ± 0.3	91.7 ± 1.8
ResNet-50	Standard	92.1	89.7
	Five-fold	91.2 ± 1.1	88.1 ± 1.0
EfficientNet-B0	Standard	96.8	97.1
	Five-fold	96.4 ± 0.3	96.2 ± 0.5
EfficientNet-B7	Standard	95.5	94.2
	Five-fold	92.0 ± 0.2	92.4 ± 1.7
RailTrack-DaViT	Standard	**99.7**	**98.8**
	Five-fold	**99.7 ± 0.1**	**98.6 ± 0.4**

**Figure A1:**
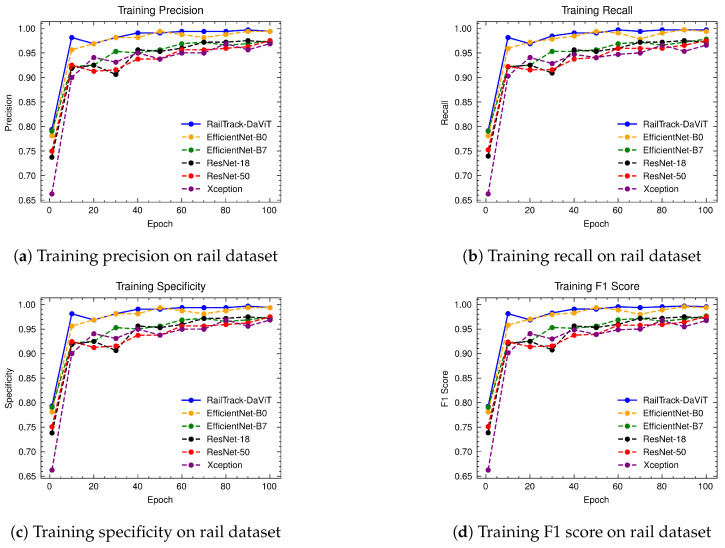
Comparative analysis of training precision, recall, specificity, and F1 score for different classification models on rail dataset over 100 training epochs.

**Figure A2:**
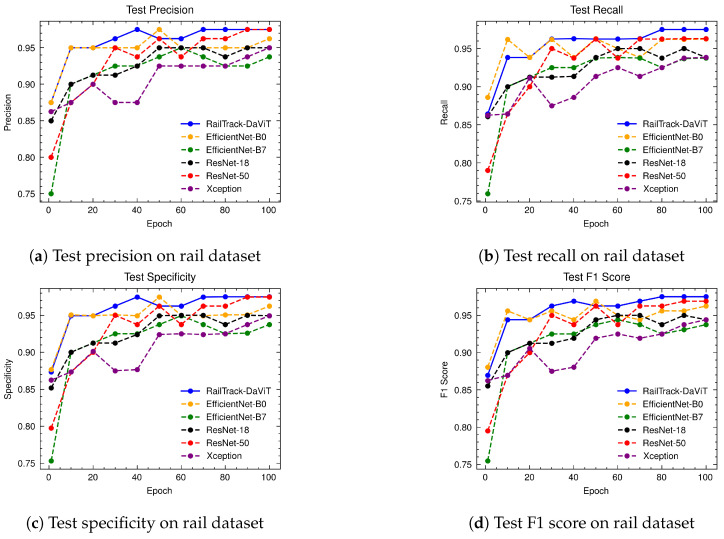
Comparative analysis of test precision, recall, specificity, and F1 score for different classification models on rail dataset over 100 training epochs.

**Figure A3:**
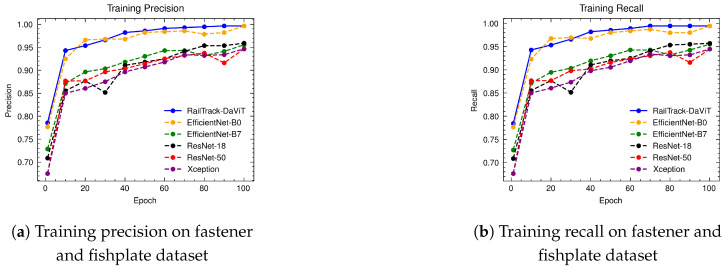

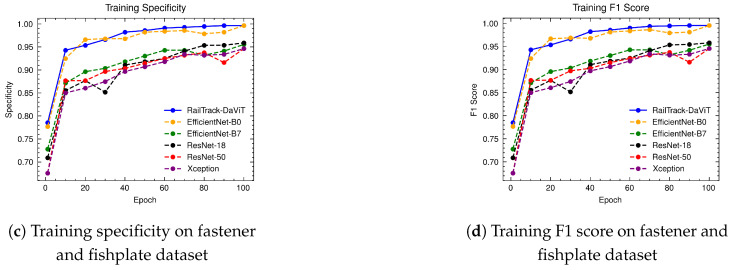
Comparative analysis of training precision, recall, specificity, and F1 score for different classification models on fastener and fishplate dataset over 100 training epochs.

**Figure A4:**
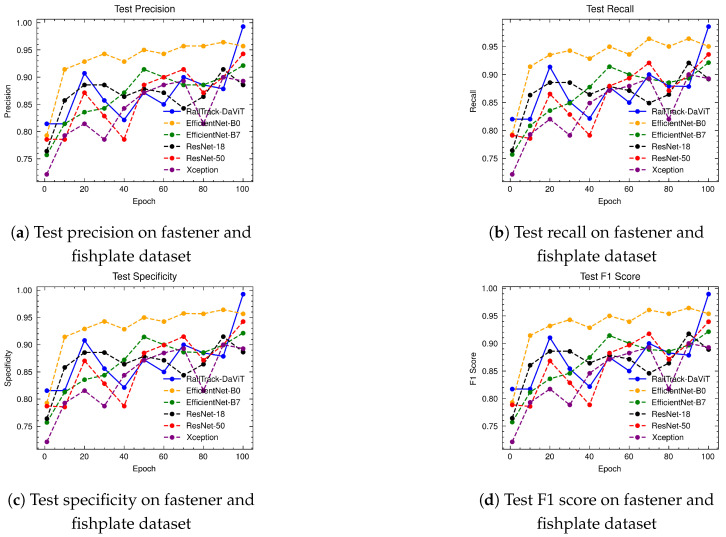
Comparative analysis of test precision, recall, specificity, and F1 score for different classification models on fastener and fishplate dataset over 100 training epochs.

**Figure A5:**
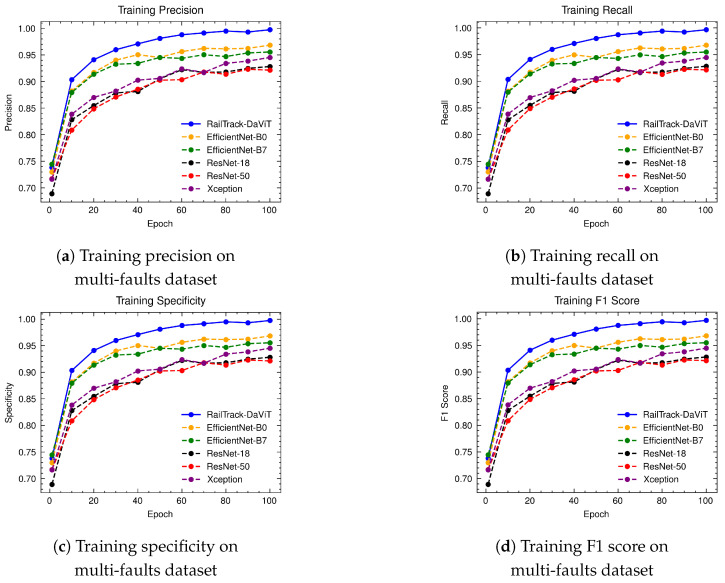
Comparative analysis of training precision, recall, specificity, and F1 score for different classification models on multi-faults dataset over 100 training epochs.

**Figure A6:**
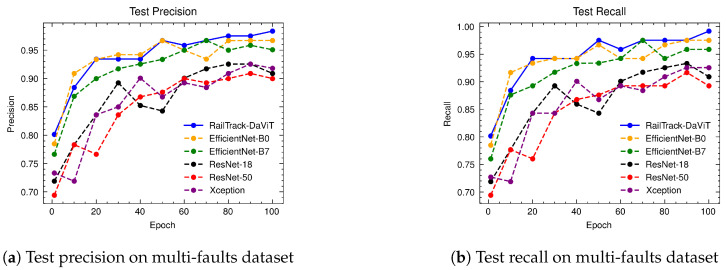

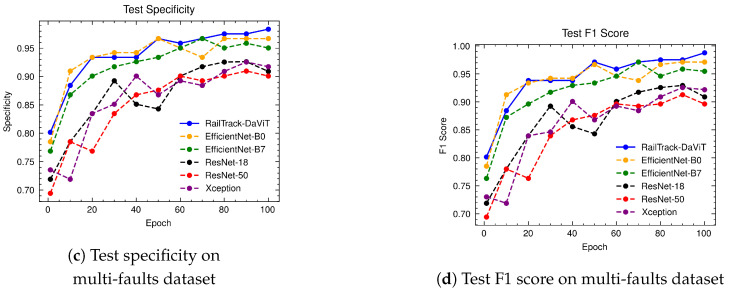
Comparative analysis of test precision, recall, specificity, and F1 score for different classification models on multi-faults dataset over 100 training epochs.

**Figure A7:**
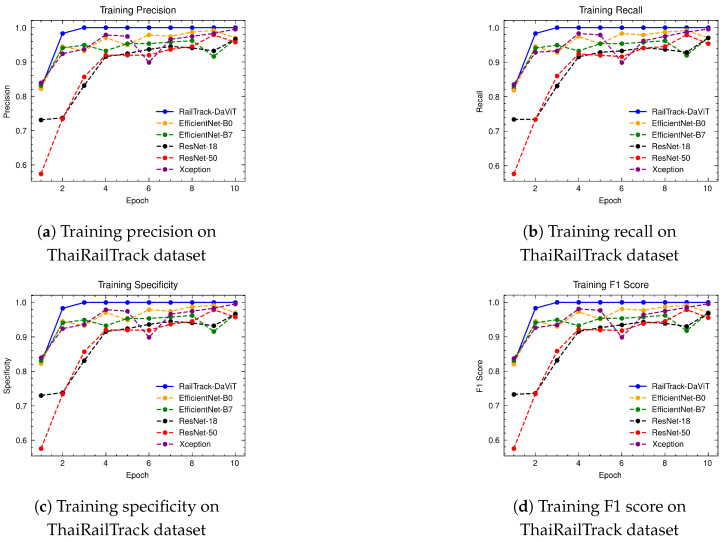
Comparative analysis of training precision, recall, specificity, and F1 score for different classification models on ThaiRailTrack dataset over 100 training epochs.

**Figure A8:**
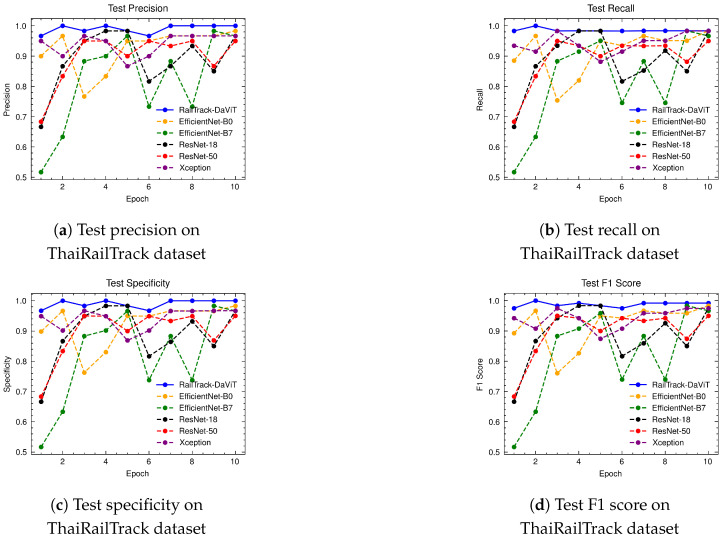
Comparative analysis of test precision, recall, specificity, and F1 score for different classification models on ThaiRailTrack dataset over 100 training epochs.

## Data Availability

Rail dataset can be found at—https://www.kaggle.com/datasets/salmaneunus/railway-track-fault-detection (accessed on 19 April 2024). Fastener and fishplate dataset can be found at—https://www.kaggle.com/datasets/ashikadnan/railway-track-fault-detection-dataset2fastener/data (accessed on 19 April 2024). ThaiRailTrack dataset is not publicly available due to privacy restrictions.

## Abbreviations

The following abbreviations are used in this manuscript:

CNN	Convolutional Neural Network
ViT	Vision Transformer
DaViT	Dual Attention Vision Transformers

## Appendix A. The Curves of Precision, Recall, Specificity, and F1 Score for Baselines and RailTrack-DaViT on Rail Dataset

Figure A1 illustrates training curves for precision, recall, specificity, and F1 score for our RailTrack-DaViT model and baseline models on rail dataset. The results demonstrate that RailTrack-DaViT and EfficientNet-B0 perform comparably; however, RailTrack-DaViT achieves the highest scores across all four metrics from the early training epochs onward.

Figure A2 illustrates test curves for precision, recall, specificity, and F1 score for our RailTrack-DaViT model and baseline models on rail dataset. The results demonstrate that RailTrack-DaViT, EfficientNet-B0, and ResNet-50 perform comparably; however, RailTrack-DaViT achieves the highest scores across all four metrics from the early training epochs onward.

## Appendix B. The Curves of Precision, Recall, Specificity, and F1 Score for Baselines and RailTrack-DaViT on Fastener and Fishplate Dataset

Figure A3 illustrates training curves for precision, recall, specificity, and F1 score for our RailTrack-DaViT model and baseline models on fastener and fishplate dataset. The results demonstrate that RailTrack-DaViT and EfficientNet-B0 perform comparably; however, RailTrack-DaViT achieves the highest scores across all four metrics from the early training epochs onward.

Figure A4 illustrates test curves for precision, recall, specificity, and F1 score of our RailTrack-DaViT model and baseline models on fastener and fishplate dataset. The results demonstrate that EfficientNet-B0 outperforms all models from the initial training state until the 90th epoch. However, starting from epoch 90, when all layers in RailTrack-DaViT were unfrozen, the performance curves of RailTrack-DaViT for all four metrics improved significantly.

## Appendix C. The Curves of Precision, Recall, Specificity, and F1 Score for Baselines and RailTrack-DaViT on Multi-Faults Dataset

Figure A5 illustrates training curves for precision, recall, specificity, and F1 score for our RailTrack-DaViT model and baseline models on multi-faults dataset. The results demonstrate that RailTrack-DaViT achieves the highest scores across all four metrics from the early training epochs onward. EfficientNet-B0 and EfficientNet-B7 rank second and third, respectively, in terms of performance.

Figure A6 illustrates test curves for precision, recall, specificity, and F1 score of our RailTrack-DaViT model and baseline models on the multi-faults dataset. The results demonstrate that RailTrack-DaViT and EfficientNet-B0 perform comparably the middle period of model training; however, RailTrack-DaViT achieves the highest scores across all four metrics in the later epochs of training.

## Appendix D. The Curves of Precision, Recall, Specificity, and F1 Score for Baselines and RailTrack-DaViT on ThaiRailTrack Dataset

Figure A7 illustrates training curves for precision, recall, specificity, and F1 score for our RailTrack-DaViT model and baseline models on ThaiRailTrack dataset. The results demonstrates that RailTrack-DaViT achieves the highest scores across all four metrics from the early training epochs onward.

Figure A8 illustrates test curves for precision, recall, specificity, and F1 score of our RailTrack-DaViT model and baseline models on ThaiRailTrack dataset. The results demonstrates that RailTrack-DaViT achieves the highest scores across all four metrics from the beginning of model training and shows slightly improvement subsequent epochs. In contrast to other CNN-based models, whose performance fluctuates during fine-tuning process on this dataset, RailTrack-DaViT exhibits more stable performance.
